# A Tale of Three Platforms: Investigating Preschoolers’ Second-Order Inferences Using In-Person, Zoom, and Lookit Methodologies

**DOI:** 10.3389/fpsyg.2021.731404

**Published:** 2021-10-13

**Authors:** Elizabeth Lapidow, Tushita Tandon, Mariel Goddu, Caren M. Walker

**Affiliations:** ^1^Department of Psychology, University of California, San Diego, San Diego, CA, United States; ^2^Department of Psychology, Harvard University, Cambridge, MA, United States

**Keywords:** developmental research, internet, research methods, cognitive development, online research

## Abstract

As a result of the COVID-19 pandemic, online methodologies for developmental research have become an essential norm. Already, there are numerous options for recruiting and testing developmental participants, and they differ from each other in a variety of ways. While recent research has discussed the potential benefits and practical trade-offs of these different platforms, the potential empirical consequences of choosing among them are still unknown. It is critical for the field to understand not only how children’s performance in an online context compares to traditional settings, but also how it differs *across* online platforms. This study offers the first comparative look at the *same* developmental task across different online research methodologies, allowing for direct comparison and critical examination of each. We conducted three versions of a test of preschoolers’ ability to generate and apply second-order inferences to predict novel outcomes. Experiment 1 is an in-person task conducted at public testing sites in the vicinity of the university. In Experiment 2, we conducted an online-moderated version of the same task, in which an experimenter presented a recording of the procedure during a live video call with families over Zoom. Finally, Experiment 3 is an online-unmoderated version of the task, in which the same videos were presented entirely asynchronously using the Lookit platform. Results suggest that online methodologies may introduce difficulties and age-related differences in young children’s performance not observed in person. We consider these results in light of the previous online developmental replications, suggest possible interpretations, and offer initial recommendations to help future developmental scientists make informed choices about whether and how to conduct their research online.

## Introduction

Much of modern behavioral psychology research is partially or entirely conducted online. The availably of survey creation software (Qualtrics, Gorilla, etc.) has enabled researchers to create digital experiments with relative ease. Online recruitment methods – including crowdsourcing platforms (Amazon Mechanical Turk, Prolific, etc.), social media, and messaging sites (Facebook, Reddit, etc.), as well as online undergraduate participant pools maintained by universities – have allowed psychologists to expand the scope and scale of their research with considerably less effort and time than traditional methods. However, this sea change has occurred almost entirely within *adult* research. Despite being notoriously hampered by time-consuming and low-return recruitment methods, developmental psychology research has remained largely in-person. There are, of course, many legitimate reasons for this. In particular, developmental methods are daunting to digitize – participants are usually too young to read written instructions or text-based stimuli, studies are often highly interactive, and many involve manipulation of physical materials. In addition, the majority of systems and software developed for online research are designed to reach audiences 18 and older. In the absence of this infrastructure and faced with such unique challenges of translation and implementation, until recently, the majority of developmental psychology was conducted entirely off-line.

The recent and rapid move of developmental research onto online platforms can be attributed to two major developments. First, over the past 5–7years, efforts to establish avenues of online research specifically designed for developmental science have begun to emerge. Researchers at MIT developed “Lookit,” the first large-scale crowdsourcing platform aimed at developmental populations and researchers (Scott et al., 2017; Scott and Schulz, 2017). Scientists can build studies within Lookit to record simple response and webcam data. These studies can then be made available to a large pool of families already registered on the Lookit website, and new families can also be invited to create accounts. At much the same time, Yale researchers launched TheChildLab.com (Sheskin and Keil, 2018), which aimed to more closely emulate traditional developmental methods by scheduling families for appointments with live researchers over video chat. During these sessions, experimenters present stimuli both verbally and visually using the video chat interface and can respond adaptively to participants and their parents in real-time.

Second, the widespread suspension of in-person activities due to COVID-19 created an urgent motivation to move developmental research online. In the last year, there has been a rapid acceleration in adoption and expansion of digital methodologies. As of early 2021, over 450 researchers from around 50 universities across seven countries were conducting research *via* Lookit,^1^ and the majority of developmental research laboratories are now actively recruiting and testing participants *via* video chat platforms. Other unmoderated systems have also emerged, including discoveriesonline.org (operated by researchers at New York University, see Rhodes et al., 2020) and themusiclab.org (operated by Harvard University). Many of these researchers have also joined with others to form the ambitious project, CRADLE (Collaboration for Reproducible and Distributed Large-Scale Experiments; see Sheskin et al., 2020), which launched the joint website, ChildrenHelpingScience.com, as a centralized resource to house listings of online developmental research studies. As of June 2021, ChildrenHelpingScience.com includes over 800 studies from laboratories all over the world, roughly a third of which are intended for children under 6years of age.

Empirical work on the validity of these platforms is still in its earliest stages, but findings published thus far are encouraging. Scott et al. (2017) conducted versions of three originally in-person experiments on Lookit, one each with infants (11–18months), toddlers (24–36months), and preschoolers (3- and 4-year olds). The latter task was a replication of Pasquini et al. (2007), which collected preschoolers’ verbal responses to investigate their sensitivity to the relative reliabilities of different informants. Although overall performance was lower on Lookit than in-person, the online study results followed the same general pattern across age groups and conditions as the original (Scott et al., 2017). In addition, Sheskin and Keil (2018) conducted several well-known developmental tasks using their video calling platform with children of different ages (5–6, 7–8, 9–10, and 11–12years). The tasks spanned different domains, including memory (for number and size), social reasoning (fairness and false-belief), and physics reasoning (gravity). Children’s answers were largely consistent with expected in-person performance, except for the false-belief reasoning task, but even in this case, the pattern of results was significant (Sheskin and Keil, 2018). In addition, researchers at New York University have conducted successful conceptual replications of older children’s in-laboratory performance using online, unmoderated testing platforms (see Leshin et al., 2021 for a replication of the effects of generic language on essentialism in 4.5–8-year olds and; Nussenbaum et al., 2020 for a replication of the development of value-learning strategies in 8–25-year olds).

Notably, however, these studies have all sought to replicate in-person performance using a single online platform. There has not, as yet, been any research that compares the same developmental study *across* platforms. The options available for conducting developmental research online differ from one another in a variety of ways, and we do not yet know what effects, if any, these differences may have on children’s performance. Many of the practical trade-offs are readily apparent (more accessible, transparent, and efficient data collection, diversifying participant demographics, lower barriers to recruitment and participation, etc.; see Sheskin et al., 2020 for review). For example, although bypassing the need for real-time experimenters means that more initial effort is required to translate traditional methodologies to asynchronous platforms, this approach also reduces the considerable time, effort, and expertise usually required for collecting developmental data.

In contrast, the potential empirical consequences of these decisions are still largely unknown. Does the presence or absence of a real-time experimenter impact young children’s engagement with an online task? If so, how should this difference in engagement be weighed against the benefits of using prerecorded procedures in ensuring consistency across participants? Questions like these will be of vital importance for developmental science in the post-pandemic world. Thus, there is a growing need for data comparing these various platforms, which can enable developmental scientists to make informed choices about whether and how to conduct their research online.

The current study offers the first comparative look at the different online research methodologies available to developmental science. We conducted three versions of the same task with preschoolers: Experiment 1 is an in-person task conducted at public testing sites in the vicinity of the university. In Experiment 2, we conducted an online-moderated version of the same task, in which an experimenter presented a recording of the procedure during a live video call with families over Zoom. Finally, Experiments 3a-c used an online-unmoderated version of the task, in which the same videos were presented entirely asynchronously using the Lookit platform. To our knowledge, this study is the first attempt to replicate the same developmental task across these three different methodologies, allowing for direct comparison and critical examination of each.

The task itself examines children’s ability to generate and apply second-order inferences to predict novel outcomes. In contrast with first-order inferences, which focus on the concrete properties of objects and events, second-order inferences capture abstract relations among those objects and events. To illustrate this, imagine looking into the window displays of two storefronts. In the one on the left, you see shirts, pants, and sweaters, and on the right, shovels, clocks, and paintbrushes. The recognition of each individual item is a first-order inference – while the realization that all of the items within a particular window belong to the same higher-order category (“*clothes*,” on the left and “*tools*,” on the right) is second-order inference.

There is some evidence that the capacity for such higher-order inferences (e.g., that boxes contain objects that are the same shape) is present even in preverbal infants (Xu and Garcia, 2008; Dewar and Xu, 2010). However, this prior work has primarily looked at infants’ *reactions* to events that are inconsistent with these second-order inferences (e.g., looking longer when a differently-shaped object is revealed). We do not know when learners begin to *utilize* these inferences to guide prediction and action. This capacity is a critical feature of second-order inferences in human reasoning. To return to our example, if you were asked which shop is more likely to sell umbrellas, you would likely be able to confidently recommend the shop on the right – despite never having observed this particular object in either window or knowing anything about the actual merchandise for sale inside.

Here, we ask whether children’s inferences about unobserved populations are sensitive to the *variability* of observed samples and whether they can use this second-order information to predict which of two hidden populations is more likely to produce a novel outcome. To test this, children watched an experimenter randomly sample balls from two identical opaque containers. The *varied-sample* consisted of four differently colored balls, and the *uniform-sample* consisted of four identically colored balls. Children were then asked which of the two containers was more likely to contain a novel-colored ball inside. If children only consider these samples in terms of their first-order properties, then we would not expect them to show a preference for either container. Considering the samples’ second-order properties, however, readily leads to an inference about the unseen populations involved. Thus, if children preferentially select the *varied-sample* container, it would demonstrate that they have not only formed this second-order inference, but also are able to use it to guide their predictions and actions beyond the limits of their direct experience.

## Experiment 1

In Experiment 1, we conduct an initial test of preschoolers’ in-person performance on a *second-order inference* task. The experimental design and analysis plan were preregistered prior to beginning data collection.^2^

### Participants

Forty children (*M*=40.12months, *SD*=5.12months, range=25.35–47.8months) were tested in Experiment 1 between November 2019 and March 2020. Participants were recruited and tested individually at local museums in a primarily urban area. While individual demographic information was not collected, demographics for recruitment locations suggest participants were predominately white (44.5%) and middle class (median household income of $73,900).

*A priori* power analysis was performed to calculate the target sample size. Our effect size (*h*=0.72) was based on results from Erb et al. (2013), which conducted a similar type of investigation (i.e., binomial analysis of a forced-choice inference question) with a similar age group. The minimum sample size needed to achieve a power of 0.8 at a significance level of 0.05 was 38, which we rounded to 40 to accommodate counterbalancing.

In addition, 13 children were tested but excluded and replaced due to experiment error (*n*=2), sibling or caretaker interference (*n*=6), or failure to respond to the test question (*n*=5).

### Stimuli

Two identical opaque containers (17''×6''×6'') were constructed from black cardboard with a cardboard egg tray concealed inside. This tray allowed the experimenter to arrange the balls inside in a specific order and then identify and draw them without looking inside the box. A felt-covered opening at the top of each box allowed the experimenter to reach inside and draw the balls one at a time.

A total of 10 plastic golf balls of different colors were also used. These balls were placed inside of each of the two containers prior to the start of the task. One container held the *varied-sample*: one green, one red, one blue, and one yellow. The other container held the *uniform-sample* of four yellow balls. In addition, both containers held one *novel-ball*, which was purple.

The task also employed two 3''×3''×8'' transparent plastic trays to hold the balls after they were drawn and a photograph of a single purple ball.

### Procedure

**Figure 1:**
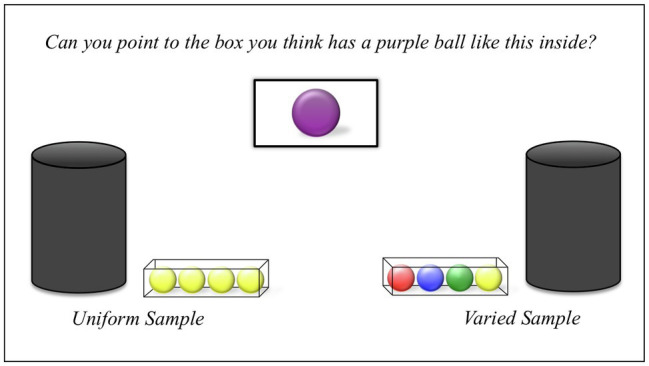
Stimuli presented to participants at test. Note that in Experiments 2 and 3, the third ball of the *varied-sample* was purple, rather than green, and the novel-ball was green, rather than purple. This was to ensure the colors would be equally distinct across different monitors.

Testing sessions began with the two opaque containers and clear trays on either side of the table (see Figure 1). The containers and trays were evenly spaced and equidistant from the participant. The experimenter told children they were going to play a game with the boxes, both of which had balls inside. She shook both containers so that the sound of the balls rattling inside was audible. The experimenter replaced the containers on the table and said, “I am going to show you some of the balls in each box,” and stepped to stand behind one of the two containers. The experimenter closed her eyes and turned her head away from the container while reaching in and pulling out a ball, apparently at random. She then directed her gaze toward the child while holding the ball out and said, “Look!,” before placing the ball into the clear plastic tray beside the container. This process of “sampling” was repeated three more times, for a total of four balls. Afterward, the experimenter repeated this process with the other container.

In this way, each participant observed a set of four balls drawn from each container. In the *uniform-sample*, all four were the same color (yellow), while in the *variable-sample*, the balls were all of different colors (one red, one blue, one green, and one yellow).^3^ The balls in the *variable-sample* were always drawn from the box in the same order. The order and side of presentation of the samples were counterbalanced across participants.

After drawing the second sample, the experimenter returned to the center of the table and addressed the child. Pointing at both the containers simultaneously, she said, “One of these two boxes has a purple ball, like this (holding a photograph of a purple ball), inside. Can you point to the box you think has the purple ball inside?” While asking this question, the experimenter looked straight ahead at the child to avoid biasing their response. If a child did not spontaneously indicate one of the two containers, the experimenter prompted by holding up the picture and repeating the question. Children who did not respond after two such prompts were excluded. After children indicated their choice, the experimenter reached into the selected container and drew a purple ball. Children were thanked for their participation and received a small gift.

### Results and Discussion

Children’s responses were recorded during the experimental session and videotaped. Response times were calculated as the time between the last word of the initial task question and when children initiated their response movement. The average response time was 8s (*SD*=7s, inter-rater reliability=90% of scores identical within +/− 1s), with only five children requiring repeated prompts to respond.

We recorded whether each child chose to search for the novel-colored ball in the *variable-sample* or the *uniform-sample* container. A significant majority of children (72.5%) chose the *varied-sample* container (*p*=0.006, two-tailed binomial). There was no significant effect of age on choice (Wald, *z*=0.881, *p*>0.378, *ns*). This suggests that young learners are not only able to form second-order inferences about the variability of unseen populations from the characteristics of observed samples, but can also apply this abstract property to guide subsequent predictions about novel events.

## Experiment 2

Having demonstrated that preschoolers succeed on this task using a traditional, in-person procedure, Experiments 2 and 3 attempted to replicate this performance online. Experiment 2 conducted the task *via* an experimenter moderated video call with participants. Using a similar approach as Sheskin and Keil (2018), families who were interested in participating were directed to sign up for appointment slots (15min each, primarily on weekend mornings) and guided through the study by an experimenter *via* video chat (Zoom).

Unlike previous work, however, the experimenter did not conduct the task herself. Instead, participants watched a video of another experimenter presenting the procedure used in Experiment 1. The “live experimenter” moderating the session controlled the playback of this video, pausing it at points when the child was asked to respond. This approach was chosen in order to maximize consistency of study delivery, which is one of the advantages of online research (e.g., Sheskin and Keil, 2018; Sheskin et al., 2020), without sacrificing the engagement and adaptability of presentation by a live experimenter. This also ensured consistency in study delivery across Experiments 2 and 3. While the ability to present online tasks in real time is a significant and potentially advantageous difference between moderated and unmoderated platforms, the goals of our investigation were best served by controlling this potential source of variation.

See Aspredicted.org for the preregistration of the experimental design and analysis plan for online replications.^4^ The video stimuli used in Experiments 2 and 3 can also be found at https://osf.io/5x8ku/?view_only=269c5468936d4811a55f237041f9ff96.

### Participants

Online participants (*N*=43, *M*=41.74months, *SD*=3.47months, range=36.2–47.9months) were tested in Experiment 2 between June and November of 2020. Children were recruited *via* email from a database of families maintained by the university’s developmental laboratories. The majority of these were families who had previously been tested and/or indicated interest in future participation at an in-person testing site. Thus, participants in Experiment 2 were from roughly the same population as those in Experiment 1. In exchange for participating, families were offered a $5.00 Amazon gift card.

An additional six children were tested, but excluded do to issues within the testing session: caretaker interference (*n*=1), failure to respond (*n*=1), or because technical issues or errors (unstable internet connection, etc.) interrupted the session (*n*=4).

### Stimuli

Testing sessions were conducted *via* the Zoom video calling platform. Three prerecorded videos (*introduction*, *test*, and *conclusion*; described below) were presented to participants using Slides.com. This meant that participants accessed the videos directly *via* their own Internet connection, leading to fewer issues of lag than screen-sharing, while still allowing the experimenter to control video playback.

The only difference in the physical stimuli between Experiments 1 and 2 was a small change in the color of the balls. In order to ensure the colors would be distinguishable across different computer monitors, two colors were switched. The purple ball was used instead of green in the *varied-sample* and the *novel-ball* and corresponding picture card were green.

### Procedure

Testing sessions began with the participating family joining the experimenter in a video call. This “live experimenter” would introduce themselves to the parent and child and then give parents an overview of the session. Families were then sent a link to the Slides.com presentation *via* the video call chat function and parents were instructed to open it and full-screen the site window.

**Figure 2:**
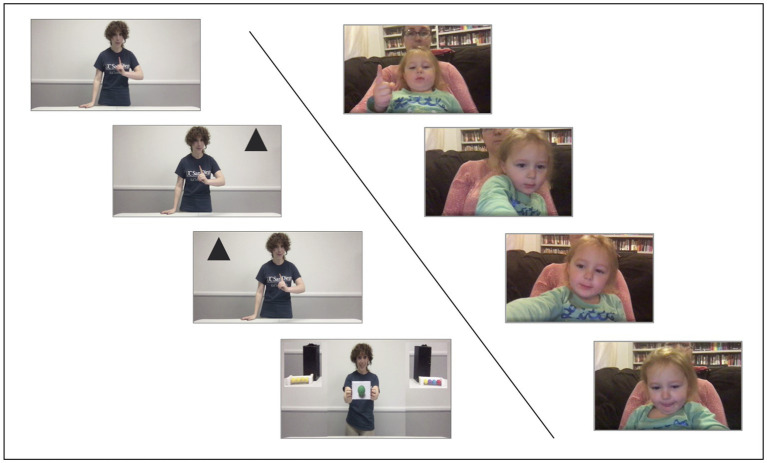
The sequence of response events in the online versions of the task. The images to the left display the pointing events from the prerecorded video shown to participants in Experiments 2 and 3. The images to the right display a sample webcam recording of the participant during each pointing event. Complete study videos may be found at https://osf.io/5x8ku/?view_only=269c5468936d4811a55f237041f9ff96.

The live experimenter would then draw the child’s attention to the screen and being playing the *introduction video*. This began with the “recorded experimenter” greeting the child and saying, “Before we start the game, let us practice using our pointing finger,” while holding her hand out in front of her with index finger extended. A black triangle would then appear in either the top right or the top left corner of the screen (added in video-editing software post recording). The recorded experimenter asked children to use their pointing finger to “touch” the black triangle (pilot testing suggested that the best way to ensure a visually distinct “right/left” point was to instruct children to get close enough to touch the upper corner of their screens). The live experimenter would pause the video playback until the child had pointed and would repeat the instructions if needed. When the video continued, the recorded experimenter said, “Good job! Let us practice one more time,” and the prompt was repeated with the triangle on the opposite side of the screen (left–right order counterbalanced across participants). This gave children a chance to practice the mode of response for the task and provided a visual calibration of what a choice for the left- or right-side container would look like (see Figure 2).

Next, the live experimenter advanced the presentation to the *test video*, in which the recorded experimenter performed the identical procedure from Experiment 1. At test, the recorded experimenter asked children to “touch” the box they thought contained the green ball. To ensure visibility of children’s responses, the images of the two boxes transitioned to the upper corners of the screen (see Figure 2). The live experimenter would pause the video until children responded. If children failed to respond spontaneously, the experimenter provided the same prompts as those used in Experiment 1. After providing a response, all children viewed a *conclusion video* in which the novel-colored ball was revealed from one container. The live experimenter then instructed parents to return to the video call window to conclude the session.

### Results and Discussion

There was no significant difference in age between the participants tested in Experiments 1 and 2, *t*(81)=−1.7, *p*=0.09 (*ns*). Children were somewhat more reluctant to respond to the task question in the moderated online platform than in person. The average response time was 11s (*SD*=18s, inter-rater reliability=91% of scores identical within +/− 1s), with 13 children requiring repeated prompts prior to responding.

The results of Experiment 2 showed a similar, but weaker pattern of performance observed in person: only 27 of the 43 children tested *via* video chat selected the *variable-sample* container (62.79%). Although this proportion was not significantly different from children’s performance in Experiment 1 (*p*=0.213, two-tailed binomial), it was also not significantly different from chance (*p*=0.126, two-tailed binomial).

*Post hoc* analysis was conducted to see whether this non-replication might be due to age-related differences in online performance. A logistic regression treating age as a continuous factor was not significant (Wald, *z*=1.271, *p*>0.204, *ns*). However, a median-split of the sample revealed that children below 3.5years of age (*n*=21, *M*=38.62months, *SD*=1.74months, range=36.2–41.5months) selected the *varied-sample* container only 52.38% of the time, which was significantly less often children 3.5years of age and older (*n*=22, *M*=44.71months, *SD*=1.49months, range=42.21–47.9months), who selected this container 72.73% of the time, *p*=0.048, two-tailed binomial).

Given that the age and general population demographics of participants were the same between Experiments 1 and 2, the difference in results appears to be due to poorer performance of the youngest children in Experiment 2. Indeed, these results are similar to those reported by Scott et al. (2017), in which 3- and 4-year-olds’ performance on Lookit was weaker than their in-person behavior, but showed the same general pattern. However, our results also suggest a developmental difference in online performance. Considering that younger children have necessarily had less experience interacting with online environments, it is possible that conducting the study online had a greater impact on their performance than older children. It is also possible that the online platform added noise equally across the age range and that younger children’s second-order inference is simply less robust.

## Experiment 3

Comparing children’s performance in Experiments 1 and 2 suggests that online tasks that require an active behavioral response (i.e., pointing) may impact performance, particularly for the youngest children. In Experiment 3, we expand this comparison to include an asynchronous online platform by using MIT’s Lookit. This platform represents a greater departure from the characteristics of traditional developmental testing than online studies conducted over video calls. Interested families create accounts on the Lookit site and are notified of studies for available for their children’s age range. The studies are composed of prerecorded and preprogrammed elements and are available for immediate participation at any time. We conducted three Lookit experiments: Experiment 3a and 3b sought to replicate the initial results with participants of the same age as those tested in Experiments 1 and 2. Then, Experiment 3c compares these results to the performance of slightly older children (4-years-old) on the same task.

### Experiment 3a

#### Participants

A total of 41 children (*M*=41.88months, *SD*=3.55months, range=36.39–47.77months) were tested *via* Lookit between November and December of 2020. Demographic information collected from parents at the time they created their accounts indicates that participants were predominately white (75%) and upper-middle class (median household income of $110,000). Families were offered a $5 Amazon gift card for their participation.

An additional 28 children were excluded or dropped. The majority were children failing to respond to the test question (*n*=9) or responding in a way that was not interpretable (e.g., pointing to the center of the screen, *n*=4). In fewer cases, children responded too late to be fully recorded (*n*=3) or children left during the videos (*n*=2). The remaining 10 exclusions were due to technical errors disrupting either the presentation of stimuli (*n*=3) or webcam recording (*n*=7).

#### Stimuli

The prerecorded videos from Experiment 2 were used in Experiment 3. These videos were embedded into the Lookit platform, which automatically displayed them in a counterbalanced order. Webcam footage was recorded during the playback of each video. Prior to the videos, written instructions with images were presented to parents to explain how to set up for recording (see *Procedure*) and what to expect within the task.

#### Procedure

Testing sessions began when parents activated the study from the listing on the Lookit page. On the first screen of the study, written instructions outlined the task. Parents were then presented with a consent document and prompted to record a verbal consent video. This was followed by an opportunity to preview the actual study videos. If a parent chose to preview, they were directed to a new screen where they confirmed their child could not see the screen (webcam footage was also recorded during this preview to later confirm the participant was not present). The preview video was a soundless, subtitled version of the task video, and presented with playback controls. All parents were then given instructions on how to set up for recording (single screen, centered webcam, etc.) and space (not backlit, faces clearly visible, minimizing distractions, etc.). Parents were instructed to have their child sit on their lap or beside them, but stressed that parents should not interact directly with their children during the game. Parents were provided with a preview of their webcam view to check that their child was visible and would be able to reach the screen, before advancing to the task itself.

A brief fixation video of a rotating ball played while webcam recording began. The task videos then played automatically. In order to ensure children had time to respond, the videos would automatically freeze for 20s at each point where children were asked to respond (i.e., twice in the *introduction video* and once in the *test video*). Parents had the option to pause the task at any time, which would transition to a separate screen showing blank screen. After the *conclusion video*, parents read a debriefing script, which explained the purpose of the study and thanked them for their participation.

#### Results and Discussion

An analysis of variance showed no significant age differences between Experiment 3a and the previous two studies, *F*(2,121)=2.315, *p*=0.103 (*ns*). The average response time was 3s (*SD*=2s, inter-rater reliability=94% of scores identical within +/− 1s). However, as this only includes children who responded within the 20-s automated timeframe (see below), this response time cannot be readily compared to those in the previous two experiments.

As in Experiment 2, children’s performance on Lookit showed a similar, but weaker trend as their in-person behavior. Overall, 26 out of 41 of children chose the *variable-sample* container at test (63.41%, *p*=0.117, two-tailed binomial). This was not significantly different from children’s performance in either Experiment 1 (*p*=0.22, two-tailed binomial) or Experiment 2 (*p*=1, two-tailed binomial). However, unlike in Experiment 2, there were no significant age differences, either when age was treated as a continuous variable (logistic regression, Wald, *z*=−1.159, *p*>0.246) or when comparing the proportion of choices for children above and below 3.5-years-old (*p*=0.269, two-tailed binomial).

The rate at which children were excluded and replaced in this experiment (28 out of a total of 69) was markedly higher than either in-person (13 out of 53) or video chat (6 out of 43). Notably, the majority of exclusions were cases in which children did not respond within the automated timeframe provided for each question. This suggests that children who were faster to respond were more likely to be included in the final sample. It is therefore possible that our failure to replicate the in-person findings (Experiment 1) or age effects (Experiment 2) was due to this potential sampling bias. In an effort to correct this, a second Lookit experiment was designed to address this aspect of the initial design.

### Experiment 3b

Although the length of response time provided in Experiment 3a (20s) was substantially longer than children’s average response times in person and on Zoom, it was insufficient for many children to respond on Lookit. In Experiment 3b, we therefore asked parents to advance the task manually after their child had responded. We also changed the implementation of webcam recordings to begin before the playback of the first video and end after the last one to capture all responses.

#### Participants

A total of 40 children (*M*=41.1months, *SD*=3.99months, range=36.07–47.80months) were tested between February and April of 2021. Participants were predominately white (65.96%) and upper-middle class (median household income of $130,000). Families were offered a $3 Amazon gift card for their participation.

A total of 12 children were excluded. Very few children failed to respond at all (*n*=2) or provided uninterpretable responses (*n*=4). The remaining exclusions were all cases of technical errors disrupting the presentation of the stimuli (*n*=6).

#### Procedure

Aside from the change in manually advance the task, the procedure for Experiment 3b was identical to Experiment 3a. The video froze following the response prompts in the *introduction* and *test videos*, and a “next” button would appear. The video would remain paused until this button was clicked. In order to ensure that parents were aware of this aspect of the task, an additional instructions screen was added. This appeared just prior to the start of the *introduction video*.

#### Results and Discussion

An analysis of variance showed no significant age differences between this and the previous experiments, *F*(3,160)=1.579, *p*=0.196 (*ns*).

The changes in task implementation in Experiment 3b reduced exclusions. In Experiment 3a, 41% of children were excluded, and majority of those exclusions were due to their failure to respond to the task question in time. In Experiment 3b, the total rate of exclusion was reduced to 23%, with no children failing to respond. This rate of exclusion was much closer to that of the in-person study (24%). There was also greater variation in response time (*M*=14s, *SD*=28s, inter-rater reliability=89% of scores identical within +/− 1s), which is unsurprising given that responses were untimed, and there was no experimenter available to prompt children to respond (see section “General Discussion” for information on rates of parental “prompts” across all experiments).

Despite these improvements, however, we failed to replicate in-person performance. Overall, children responded at chance (57.5%, *p*=0.43, two-tailed binomial). This result was not different from performance in Experiment 2 (*p*=0.515) and Experiment 3a (*p*=0.512) and only marginally different from Experiment 1 (*p*=0.049, two-tailed binomial). As in Experiment 3a, there was no effect of age when treated as a continuous variable (logistic regression, Wald, *z*=0.538, *p*>0.591) or when comparing the proportion of *varied-sample* choices between younger and older children (*p*=0.521, two-tailed binomial).

These results rule out the possibility that the lower performance observed in Experiment 3a was due to the time constraint on responses. However, it is unclear whether the lack of replication is due to an increased difficulty with the online task or whether the unfamiliar testing platform impeded children’s second-order inferences. It is also possible that this digital, prerecorded context led children to treat the two samples as equivalent. In order to distinguish among these possibilities, we examine the online performance of slightly older children in Experiment 3c.

### Experiment 3c

In an effort to identify what caused children’s chance performance in the unmoderated online testing platform, we conducted another study on Lookit with an older sample of children. The results of Experiment 2 suggest that children’s online performance on this second-order inference task may become more robust with age. If so, then older children’s performance on an unmoderated online platform should be more likely to resemble in-person performance.

#### Participants

A total of 42 children (*M*=55.05months, *SD*=3.04months, range=48.23–59.57months) were tested during April of 2021. Demographic information indicated participating families were predominately white (61.36%) and middle class (median household income of $100,000). Families were offered a $3 Amazon gift card for their participation. Two additional children were excluded: one for inattention during the task videos and one for observing the study preview.

#### Method

The stimuli and task procedure were identical to Experiment 3b.

#### Results and Discussion

**Figure 3:**
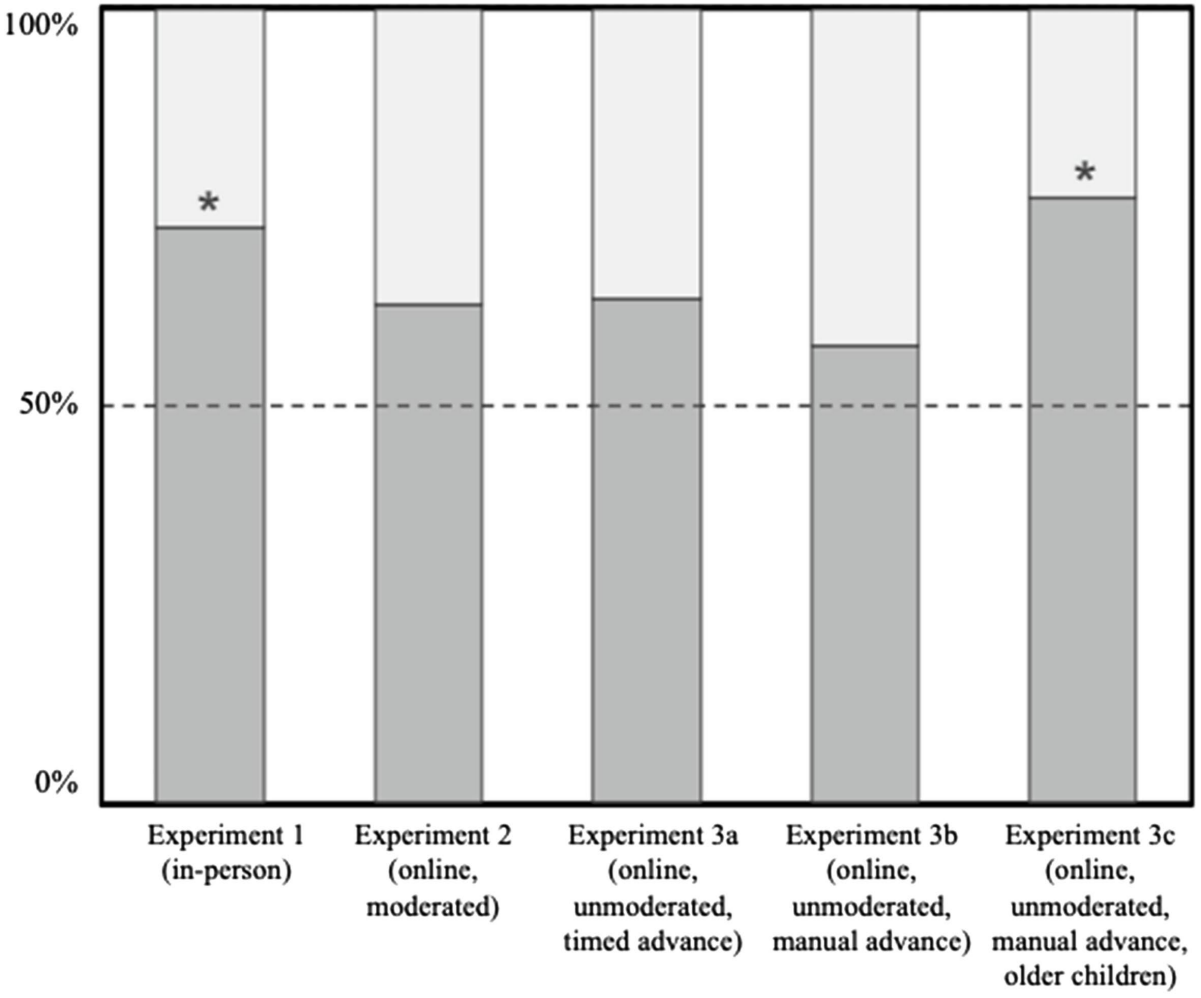
The proportion of children’s choices across in-person (Experiment 1), online-moderated (Experiment 2) and online-unmoderated (Experiments 3a-3c) testing platforms. Asterisks denote significance at *p*<0.05.

Of the 42 4-year olds tested in Experiment 3c, 32 selected the *varied-sample* container (76.19%). Performance was greater than in Experiment 3b (*p*=0.018) and marginally greater than in Experiment 2 (*p*=0.08), but not different from either Experiment 1 (*p*=0.73) or Experiment 3a (*p*=0.108, two-tailed binomial). See Figure 3 for a comparison of children’s performance across all experiments and platforms. As in Experiment 1, children choose the *varied-sample* significantly more often than chance (*p<* 0.001, two-tailed binomial).

Four-year-olds also responded much more readily to the task question, with an average response time of 4s (*SD*=6s, inter-rater reliability=97% of scores identical within +/− 1s), with no children failing to respond. Rates of parental involvement were also lower (see section “General Discussion”).

These findings suggest that children are not making a genuinely different inference due to the online presentation of the study, but that younger children’s ability to generate and act on their inference may be less robust online than in-person.

## General Discussion

While still in their early stages, online platforms and protocols are poised to become a normalized and valuable part of developmental science. The COVID-19 pandemic forced researchers to meet the challenges of translating their studies into digital, distanced methodologies. Having overcome this initial hurdle, it is very likely that researchers will continue to utilize online methodologies after the return to in-person testing. The potential of online recruitment for accessing larger, more diverse, and lower-effort sources of developmental participants, as well as the ease of transparency, collaboration, and reproducibility of online protocols will continue to offer compelling opportunities for developmental science well into the post-pandemic world (Scott et al., 2017; Sheskin and Keil, 2018).

The current study offers an early, comparative look into how these possibilities might be realized across different online developmental research methods. We conducted the same second-order inference task with preschoolers in a traditional, in-person research setting (Experiment 1), *via* moderated video chat (Zoom; Experiment 2), and *via* an unmoderated, crowdsourcing site (Lookit; Experiments 3a–c). Figure 3 compares children’s performance across all experiments and platforms. In all versions of the task, the majority of children selected the *varied-sample* container, but this pattern of performance was weaker online. In both moderated and unmoderated online platforms, only the oldest children’s (3.5–4-year old on Zoom and 4–5-year old on Lookit) choices were different from chance.

Considering our results in light of the previous findings suggests possible interpretations and recommendations for future developmental research. First, we were unable to fully replicate the children’s successful in-person performance on a forced-choice second-order inference task in either moderated or unmoderated online platforms. This contrasts with previous research that has successfully replicated other in-person developmental results online. However, much of that work involved older children (e.g., Sheskin and Keil, 2018; Leshin et al., 2021) or implicit looking-time measures with infants (e.g., Scott et al., 2017). There is extensive evidence that children’s success on looking-time measures precedes their ability to act in numerous domains (e.g., Zelazo et al., 1996; Hood et al., 2003; Kirkham et al., 2003). As such, it is perhaps unsurprising that preschoolers’ performance on our task, which required an explicit response based on a second-order inference, was too fragile to translate online. We believe that these results should be treated as informative, rather than prohibitive, for future online research. They suggest that studies involving an explicit response from young children may be particularly challenging to conduct online, unless performance is expected to be particularly robust.

Notably, this research was conducted sequentially, rather than simultaneously, and this timing should be taken into account when interpreting the results. As noted in above, data collection for Experiment 1 was completed in early 2020, just prior to the stay-at-home orders due to COVID-19. All online testing was conducted over the course of the next 14months: Zoom data collection for Experiment 2 began in the summer of 2020 and concluded in late fall, while the Lookit studies were conducted in late 2020 and early 2021. Thus, our data were collected during rather distinct periods of social and societal change. It is therefore difficult to speculate what impact these changes may have had on our results, especially since children’s online performance does not seem to have improved with the dramatic increase in exposure to online platforms during this time.

This study also offers novel insights into the nature of online testing across different platforms, which may suggest important points of consideration for future research. For example, we found that parents were more inclined to interact and engage with their children during online testing sessions. Note that preregistered exclusion criteria prevented any potential impact of parental *interference* (e.g., a parent pointing at the stimuli). Non-interference interactions included neutral prompts and encouragements to respond (e.g., “Which one do you think it is?” “Can you point now?”). While these interactions were much more common during Zoom and Lookit testing than in-person (Experiment 1, *n*=3), there was not much difference between the synchronous (Experiment 2, *n*=17) and asynchronous untimed sessions (Experiment 3b, *n*=20) for 3-year-old children. The rate of parental interactions was lower in Experiment 3a (*n*=9), likely due to the limited response window, and in Experiment 3c (*n*=10), which was conducted with 4-year-olds. This increased parental involvement has potential to be beneficial, as it may help to reduce attrition during asynchronous testing. However, researchers should provide instructions to parents to control this interaction, and treat this aspect of the experiment as part of the study design.

Similarly, future researchers should make careful efforts to capitalize on the potential for online testing to broaden and diversify developmental participant pools. The current study did not attempt to control the demographics of Lookit participants and Experiments 3a–3c ultimately included *more* affluent, *less* diverse samples than those in Experiments 1 or 2. This will not only help to ensure the quality and comparability of online developmental data, but also to take advantage of the recruitment opportunities these platforms afford.

Finally, the current study highlights the potential use of online platforms for facilitating nuanced methodological and developmental comparisons. The time, effort, and cost of collecting developmental data often prohibit including additional comparison and control groups – even when this is the recommended approach. In the current study, we were able to conduct an identical version of a previous experiment with older children in order to clarify the developmental trajectory of children’s performance on our task, with ease. While every effort was made to ensure the consistency of the procedure in Experiment 1, it was ultimately easier to achieve this consistency in Experiments 2 and 3. However, given the increased parental interaction, there was also some variability in the online procedures. Additionally, the period of data collection for the unmoderated online experiments (1–2months) was less than half that of Experiments 1 and 2 (~5months).

This study, along with the others in this special issue, represents some of the very first steps in better understanding the process, pitfalls, and potential of taking developmental research online. We hope that our results will serve to encourage and empower the field of developmental science to make the best possible use of these new methods going forward.

## Data Availability Statement

The datasets presented in this study can be found in online repositories. The names of the repository/repositories and accession number(s) can be found at: https://osf.io/5x8ku/?view_only=269c5468936d4811a55f237041f9ff96.

## Ethics Statement

The studies involving human participants were reviewed and approved by the University of California, San Diego, Human Research Protections Program. Written or video-recorded informed consent to participate in this study was provided by the participants’ legal guardian/next of kin. Written informed consent was obtained from the individual(s) for the publication of any identifiable images or data included in this article.

## Author Contributions

EL and MG developed the concept, hypothesis, and design for the study. TT conducted the investigation and data curation, and drafted the manuscript. EL conducted the analysis and drafted and revised the manuscript. CW supervised the study, helped to develop the hypotheses, and revised the manuscript. All authors contributed to the article and approved the submitted version.

## Funding

This study was supported by funding from the National Science Foundation (CAREER grant #2047581), Hellman Foundation, Jacobs Foundation Fellowship, and the National Defense Science and Engineering Graduate Fellowship.

## Conflict of Interest

The authors declare that the research was conducted in the absence of any commercial or financial relationships that could be construed as a potential conflict of interest.

## Publisher’s Note

All claims expressed in this article are solely those of the authors and do not necessarily represent those of their affiliated organizations, or those of the publisher, the editors and the reviewers. Any product that may be evaluated in this article, or claim that may be made by its manufacturer, is not guaranteed or endorsed by the publisher.

## Footnotes

^1^ https://lookit.mit.edu/scientists/

^2^ https://aspredicted.org/blind.php?x=rb4jn6

^3^Samples were selected based on the procedure used by Sim and Xu (2013).

^4^ https://aspredicted.org/blind.php?x=t85n33
